# Properties of cellular and serum forms of thymidine kinase 1 (TK1) in dogs with acute lymphocytic leukemia (ALL) and canine mammary tumors (CMTs): implications for TK1 as a proliferation biomarker

**DOI:** 10.1186/s12917-014-0228-1

**Published:** 2014-10-08

**Authors:** Kiran Kumar Jagarlamudi, Sara Westberg, Henrik Rönnberg, Staffan Eriksson

**Affiliations:** Department of Anatomy, Physiology, and Biochemistry, Swedish University of Agricultural Sciences, Biomedical Center, P.O. Box 575, S-751 23 Uppsala, Sweden; University Animal Hospital, Swedish University of Agricultural Sciences, P.O. Box 7040, S-750 07 Uppsala, Sweden; Center of Clinical Comparative Oncology (C3O), Department of Clinical Sciences, Swedish University of Agricultural Sciences, P.O. Box 7054, S-750 07 Uppsala, Sweden

**Keywords:** Canine serum thymidine kinase 1, sTK1 protein assays, Canine mammary tumors, Size exclusion chromatography, Anti-dog TK1 antibodies

## Abstract

**Background:**

Thymidine kinase 1 (TK1) is a deoxyribonucleic acid (DNA) precursor enzyme and a proliferation biomarker used for prognosis and treatment monitoring of breast cancer in humans. The aim was to determine if serum thymidine kinase 1 (sTK1) activity and sTK1 protein levels in dogs with mammary tumors could be useful in veterinary medicine.

**Results:**

Serum samples from 20 healthy dogs and 27 dogs with mammary tumors were analyzed for sTK1 activity, using an [^3^H]-deoxythymidine (dThd) phosphorylation assay, and for sTK1 protein levels by immune affinity/Western blot assay. The molecular forms of sTK1 in acute lymphocytic leukemia (ALL), canine mammary tumor (CMT), and healthy sera were determined by size exclusion chromatography.

Mean sTK1 activities in CMT were 1.0 ± 0.36 pmol/min/mL, differing significantly from healthy dogs (mean ± SD = 0.73 ± 0.26 pmol/min/mL). Serum TK1 protein (26 kDa polypeptide) levels were also significantly higher in CMTs compared to healthy dogs (mean ± SD = 28.5 ± 11.4, and 8.5 ± 4 ng/mL, respectively). Cellular TK1 isolated from ALL tumor cells was predominantly a dimer, while the serum TK1 activity eluted as a high molecular weight (MW) oligomer. In analyses of CMT tissue extracts, TK1 activity eluted in two peaks, a minor peak with a high MW oligomer and a major tetramer peak. Western blot analysis of chromatographic fractions showed that cellular TK1 protein in both ALL and CMT dogs, and to some extent serum TK1 from ALL dogs, correlated with activity profiles, but a large fraction of inactive TK1 protein was detected in CMT.

**Conclusions:**

Serum TK1 protein and activity levels were significantly higher in CMT than in healthy dogs. Size exclusion chromatography demonstrated major differences in the molecular forms of sTK1 in ALL, healthy, and CMT dogs, with a large fraction of inactive TK1 protein in CMT. Our results showed that the sTK1 protein assay can differentiate benign tumors (early stage tumors) from healthy more efficiently than sTK1 activity assay. This preliminary data supports that sTK1 protein assay is clinically useful. Further studies are needed to evaluate the diagnostic or prognostic role of serum TK1 protein in CMTs.

## Background

Mammary tumors are the most common form of canine tumors and account for approximately 50% of all tumors in female dogs [1,2]. Development of canine mammary tumors (CMTs) is influenced by age, hormone and genetic predispositions. CMTs are commonly seen in bitches at middle and old age, with a median age of 8–10 years [3-5]. Purebreed dogs have higher incidence of CMT [6] and breeds like Poodles, English Springer Spaniels (ESSs), German Shepherds, Boxers, and Dachshunds have a higher risk of developing CMT [1]. Overall, human and dog cancer diseases show similarities in terms of the role of steroidal hormones during tumor development, and correlation between tumor grade and rate of metastasis [7,8]. A recent study has shown that variations in the BRCA1 and BRCA2 genes are associated with CMT in ESSs [9] and these genes are responsible for early onset of breast cancer in women. Inherited mutations in BRCA1 or BRCA2 increase the risk of breast cancer in women by around 56–84% [10,11]. The clinical impact and outcome of having BRCA mutations is somewhat unclear in humans [12-15]. BRCA1-associated breast cancers often occur in younger women, and these tumors are high grade and lack estrogen receptors (ERs), and are often triple-negative and, as such, harder to treat [16]. In dogs, overexpression of BRCA1 and BRCA2 has been found in mammary adenocarcinomas and their lymph node metastases, hence implying a tendency to reflect grade of malignancy [17]. Nevertheless, comparative breast cancer studies are likely to greatly benefit both human and veterinary medicine.

Early detection of CMTs and efficient therapy can increase the life span in dogs [18-20]. Several tumor markers, such as carbohydrate antigen 15–3 (CA 15–3), thymidine kinase 1 (TK1), carcinoembryonic antigen (CEA), estrogen receptor (ER), and human epidermal growth factor receptor 2 (HER-2/neu), have been evaluated for their capacity to aid in the diagnosis, prognosis, and monitoring of breast cancer in humans [21-24]. Some of these markers, like CA 15–3, CEA, ER, and HER–2, have been clinically evaluated in dogs [25-27], but investigations on TK1 in CMTs are largely lacking.

Thymidine kinase (TK) is a cytosolic enzyme that catalyzes the conversion of deoxythymidine (dThd) to deoxythymidine monophosphate (dTMP), which is subsequently phosphorylated to di- and triphosphates via the salvage pathway [28]. Thymidine kinase 1 is cell cycle-dependent and S phase-specific. The TK1 activity reaches a peak G1/S boundary and then decreases rapidly in G2 and becomes undetectable in M phase [29,30]. In human medicine, serum TK1 (sTK1) activity level serves as biomarker for prognosis and monitoring treatment of lymphoma and leukemia [31-33]. Serum TK1 activity has been determined by using the commercially available TK-radioenzyme assay (REA) [34] and non-radiometric TK-Liaison assay [35]. These activity-based assays provide valuable information regarding prognosis and treatment monitoring in canine lymphoma and leukemia [35,36]. A recent study on sera from dogs with solid tumors demonstrated that determination of sTK1 protein levels with immunochemical methods gave higher sensitivity compared to TK1 activity measurements [37].

To date, no studies exist where both TK1 activity and TK1 protein levels have been determined in sera from CMT patients. In this study, we analyzed TK1 activity and TK1 subunit 26 kDa protein levels in sera from 27 dogs with CMTs. We further characterized the molecular forms of TK1 in ALL, CMT, and healthy dog sera using size exclusion chromatography. In the case of ALL, it was possible to study the cellular form of TK1 isolated from the leukemic cells, as in the CMT tissue extract. To our knowledge, this is the first time that cellular and serum forms of TK1 from the same patient have been determined.

## Methods

### Serum samples

Sera from healthy dogs and dogs with mammary tumors were collected from the University Animal Hospital at the Swedish University of Agricultural Sciences, Uppsala, Sweden, and stored at −20°C until analysis. The project was approved by the Swedish Animal Ethics Committee and owner consent was collected for each patient. The study comprised samples from healthy dogs (n = 20) and dogs with mammary tumors (n = 27: benign n = 11, malignant n = 13, and unclassified (UC) n = 3). The mean and median age was 6 years (range 3–10 years) for the healthy group, and 9.5 and 9 years (range 3–14 years) for dogs with mammary tumors.

The tumor diagnostic procedures were as described previously [35]. A total of 20 breeds were included in this study. The most common breeds included were Labrador Retriever, Rottweiler, Boxer, and German Shepherd (5/47 of each), followed by Golden Retriever and Riesenschnauzer (4/47 each) Rhodesian Ridgebacks and mixed breed (3/47 of each), Bernese Mountain Dog and ESS (2/47 of each), Field Spaniel, Flatcoated Retriever, Border Collie, American Cocker Spaniel, Welsh Springer Spaniel, Belgian Shepherd, Dalmatian, Dachshund, and Greyhound (1/47 of each). Polyclonal antibodies were produced against different epitopes in the C-terminal region of dog TK1. The 28-mer antibody was produced using a 28 amino acid synthetic peptide (amino acids 195–223) as antigen (Agrisera AB, Umeå, Sweden). The 16-mer antibody was produced against a 16 amino acid C-terminal region (amino acids 211–225) (GenScript, Piscataway, NJ, USA) [38].

### Isolation of acute lymphocytic leukemia cells and preparation of cell extract

An amount of 4 ml of heparinized ALL patient blood was diluted with an equal part of sterile phosphate-buffered saline (PBS) and the diluted blood sample was mixed with an equal volume of Ficoll-Paque. This was followed by centrifugation for 30 minutes at 1,500 revolutions per minute (RPMs). The cell band was removed, and washed three times with RPMI 1640 medium with L-glutamine containing 20% fetal calf serum (FCS) and 50 μg/ml gentamicin. The cell preparation contained at least 95% ALL cells with a viability of >98%. Cell viability was estimated by using the Trypan blue exclusion assay. If the cells take up trypan blue it indicates that the cells are non-viable. Cell viability should be at least >95% for healthy log-phase cells in cultures. Percentages of viable cells were determined by using the following formula:

$$ \%\ \mathrm{viable}\ \mathrm{cells} = \left[1.00\ \hbox{--}\ \left(\mathrm{Number}\ \mathrm{of}\ \mathrm{blue}\ \mathrm{cells} \div \mathrm{Number}\ \mathrm{of}\ \mathrm{total}\ \mathrm{cells}\right)\right] \times 100. $$

Cells (16 × 10^6^) were lysed in buffer containing 10 mM Tris/HCl, pH 7.6, 13.7 mM NaCl, 7 mM ethylenediaminetetraacetic acid (EDTA), 0.5% NP-40, and 2 mM 4-2(aminoethyl)benzenesulfonyl fluoride hydrochloride (Pefabloc, Fluka, Seelze, Switzerland) for 30 minutes at 4°C. The suspension was centrifuged at 13,000 RPMs for 10 minutes at 4°C and supernatant was stored in aliquots at −80°C after addition of 20% of glycerol. Protein concentration in the supernatant (4 mg/mL) was determined using the Bio-Rad assay.

### Extraction of thymidine kinase 1 from canine mammary tumor tissue

Three grams of fresh tumor tissue (from dog No. 23) were collected from the clinic in sterile PBS, pH 7.6. The accuracy of the tumor diagnosis was confirmed by histopathological analysis of two flanking tissue preparations right adjacent to the tissue used for TK1 analysis. The TK1 enzyme extract was prepared as follows: The tissue was homogenized in a Teflon homogenizer with two volumes of 0.05 M Tris-HCI buffer, pH 8.0, containing 0.34 M sucrose, 25 mM KCI, 5 mM MgCI2, 7 mM EDTA, 0.5% NP-40, and 2 mM 4-2(aminoethyl)benzenesulfonyl fluoride hydrochloride (Pefabloc, Fluka, Seelze, Switzerland). The homogenate was centrifuged at 20,000 RPMs for 30 minutes. The supernatant with a protein concentration of 3 mg/mL was stored at −20°C for further analysis.

### Serum thymidine kinase activity assay

Thymidine kinase 1 activity in serum samples was determined using an optimized [^3^H]-dThd phosphorylation assay, as described previously [36]. In brief, this assay is based on a radio enzymatic technique in which the substrate ^3^[H]-thymidine is converted to ^3^[H]-thymidine monophosphate by TK1 in serum. The TK1 activity is expressed as pmol of dTMP formed per minute per mL of serum.

### Immunoaffinity Sepharose preparation

The purified polyclonal anti-dog TK1 antibody (16-mer) was coupled to cyanogen bromide (CNBr)-activated Sepharose 4B, as described previously [37]. Briefly, 2 mg of purified 16-mer antibody was added per 1 g of the CNBr-activated Sepharose 4B resin in 0.1 M sodium bicarbonate (NaHCO_3_, pH = 8.3) on agitation for 4 hours at room temperature. Then the coupled Sepharose was transferred into Tris–HCl buffer (0.1 M, pH 8.0) after 2 hours at room temperature. The resin was washed alternately three times with acetate buffer (0.1 M, pH 4.0) and Tris–HCl buffer (0.1 M, pH 8.0). The coupled antibody-Sepharose was mixed with Tris-buffered saline (TBS) containing 0.01% NaN_3_ 1:1, and stored at 4°C.

### Size exclusion chromatography

Size exclusion chromatography was performed as described elsewhere [38,39] using a Superose 12 column (1.0 × 30 cm; GE Healthcare, Uppsala, Sweden) attached to fast protein liquid chromatography (FPLC) equipment (GE Healthcare, Uppsala, Sweden). Acute lymphocytic leukemia cell extract, ALL sera (100 μl), CMT tissue extract, CMT sera (from dog No. 23), and sera from a healthy dog (No. 5) (200 μl) were diluted in hydroxyethyl-piperazineethane-sulfonic acid (HEPES), pH 7.6, buffer (0.01 M) (NH_4_Cl, 0.15 M, and NaN_3_, 0.02%) and applied on the column. Standard proteins were α2-macroglobulin, 720 kDa; β-amylase, 200 kDa; bovine serum albumin (BSA), 66 kDa; ova albumin, 45 kDa; and horse myosin, 17 kDa.

### Thymidine kinase 1 (TK1) isolation by anti-TK1 antibody Sepharose

Dog recombinant TK1 (0.5–4 ng), 60 μL, serum samples were diluted with TBS, pH 7.6, to a final volume of 230 μL. Next, 300 μL of FPLC fractions were incubated with anti-dog TK1 antibody Sepharose (70 μL of a 1:1 V/V mixture of antibody Sepharose and TBS). The samples were agitated at 4°C for 4 hours, followed by centrifugation for 1 minute at 13,000 RPMs. The antibody Sepharose was washed twice with TBS, once with TBS-Tween, and once more with TBS. Then, 30 μL of sodium dodecyl sulfate polyacrylamide gel electrophoresis (SDS-PAGE) sample buffer (containing Tris–HCl, pH 6.8, 0.5 M; glycerol, 20%; (w/v) SDS, 10%; bromophenol blue, 0.1%; and dithiothreitol (DTT), 10 mM) was added to the Sepharose before incubation at room temperature for 20 minutes, followed by centrifugation as above. The supernatants were heated at 95°C for 5 minutes and subjected to 12% SDS-PAGE, followed by electrophoretic transfer and Western blotting, as described previously [37,38].

### Statistical analysis

The TK1 activity and protein distribution in both groups were tested for normality using the D’ Agostino and Pearson omnibus normality test. The majority of distributions were Gaussian and we used one way analysis of variance (ANOVA) followed by Tukey’s multiple comparison post test to compare TK1 values across multiple groups. Pearson correlation coefficient (*r)* was used to determine the correlation between TK1 activities and TK1 protein levels. Unpaired *t-*test was used to evaluate the difference between the groups. For sensitivity and specificity analysis and for comparison of assays, receiver operating characteristic (ROC) curves were constructed. Statistical analyses were performed using Graph Pad Prism 5.0 (Graph Pad Software, La Jolla, CA, USA). The level of significance was set at P < 0.05.

## Results

### Serum thymidine kinase 1 activity and protein levels in canine mammary tumor

**Figure 1:**
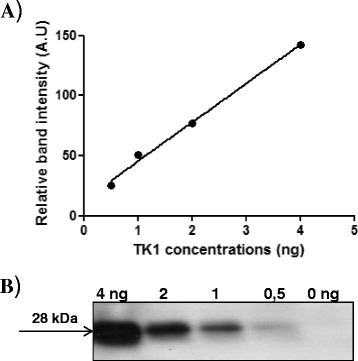
**Standard curve for recombinant TK1 protein using the immune affinity assay and Western blot analysis. (A)** Concentration curve obtained after scanning the relative band intensities (arbitrary units (AUs)). **(B)** The 28 kDa polypeptide bands of recombinant dog thymidine kinase 1 (TK1) (0.5–4 ng).

**Figure 2:**
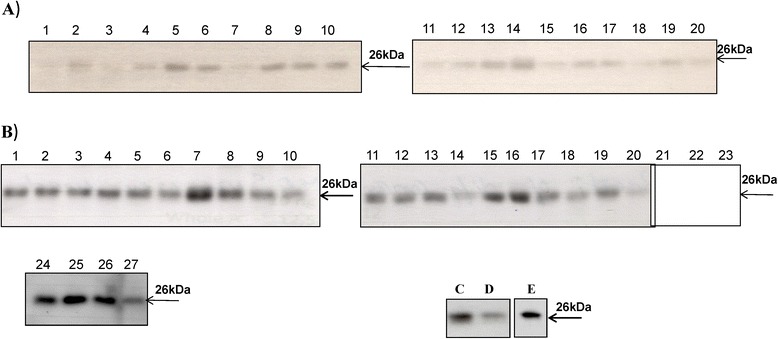
**Immunoaffinity and Western blot results for sera from healthy dogs (A), dogs with canine mammary tumors (CMTs) (B), and a dog with acute lymphocytic leukemia (ALL) (C).** The Figure shows the TK1 band determined in the ALL cell extract **(D)** and a CMT (M-23) tissue extract **(E)**.

Sera from 20 healthy dogs and 27 CMT dogs were analyzed for sTK1 activity and sTK1 protein, as described in the Materials and Methods section. The intensities of the 28 kDa recombinant dog TK1 polypeptide (0.5–4 ng) were analyzed and a standard curve was created, as shown in Figure 1. Serum TK1 protein levels in clinical samples were calculated using this curve. In the healthy group, sTK1 activity was in the range of 0.4–1.4 pmol/min/mL, with a mean ± standard deviation (SD) of 0.73 ± 0.26 (Table 1). In the immunoaffinity/Western blot analysis, faint bands were detected in sera from healthy dogs, with a range of 3–18 ng/mL (mean ± SD of 8.5 ± 4 ng/mL; Figure 2A). In the CMT group, the sTK1 activity was 0.5–2.5 pmol/min/mL (mean ± SD = 1.0 ± 0.36). High sTK1 protein levels were found in the mammary tumor sera (Figure 2B) and the sTK1 protein concentration ranged from 9 ng/mL to 54 ng/mL (mean ± SD of 28.5 ± 11.4; Table 2). Serum TK1 protein levels in the ALL serum (36 ng/mL; Figure 2C), ALL extract (20 ng/mL; Figure 2D), and CMT extract (34 ng/mL; Figure 2E) were also determined.

**Table 1:** **Serum thymidine kinase 1 (sTK1) activity and sTK1 protein concentration in sera from healthy dogs**

			**sTK1 activity**	**sTK1 concentration**
**Number**	**Breed**	**Age (yrs)**	**(pmol/min/mL)**	**(ng/mL)**
			**(Mean ± SD)** ^**a**^	**(Mean ± SD)** ^**b**^
1	Labrador Retriever	8	0.7 ± 0.03	3 ± 1
2	Bernese Mountain Dog	10	1.0 ± 0.05	9 ± 3
3	Labrador Retriever	5	0.5 ± 0.04	4 ± 2
4	Golden Retriever	6	0.4 ± 0.02	7 ± 3
5	Golden Retriever	5	1.0 ± 0.06	15 ± 4
6	German Shepherd	6	1.0 ± 0.1	11 ± 3
7	Riesenschnauzer	7	0.5 ± 0.02	4 ± 2
8	Mixed breed	6	0.5 ± 0.05	12 ± 5
9	Riesenschnauzer	8	0.6 ± 0.04	10 ± 4
10	German Shepherd	6	0.7 ± 0.08	12 ± 3
11	Rottweiler	5	0.5 ± 0.03	7 ± 3
12	English Springer Spaniel	3	1.3 ± 0.09	8 ± 2
13	Riesenschnauzer	7	1.4 ± 0.12	13 ± 4
14	Labrador Retriever	5	0.8 ± 0.07	18 ± 5
15	Labrador Retriever	6	0.7 ± 0.03	5 ± 3
16	Mixed breed	6	0.6 ± 0.01	9 ± 2
17	Bernese Mountain Dog	5	0.7 ± 0.04	8 ± 3
18	Dalmatian	6	0.5 ± 0.03	4 ± 2
19	Labrador Retriever	4	0.7 ± 0.11	8 ± 3
20	Golden Retriever	6	0.5 ± 0.04	6 ± 2

^**a**^Mean values of three observations from a single measurement occasion. ^**b**^Mean values of two observations from two independent experiments. SD = standard deviation.

**Table 2:** **Serum thymidine kinase 1 (sTK1) activity and protein levels in sera from dogs with mammary tumors**

				**sTK1 activity**	**sTK1 concentration**
**Number**	**Breed**	**Age (yrs)**	**Diagnosis**	**(pmol/min/mL)**	**(ng/mL)**
				**(Mean ± SD)** ^**a**^	**(Mean ± SD)** ^**b**^
1	Field Spaniel	9	Mammary adenoma	0.8 ± 0.04	26 ± 4
2	Rhodesian Ridgeback	11	Mammary adenoma	0.9 ± 0.03	26 ± 5
3	Rhodesian Ridgeback	3	Mammary adenoma	0.6 ± 0.02	23 ± 3
4	Boxer	13	Mammary carcinoma	0.9 ± 0.04	27 ± 2
5	American Cocker Spaniel	11	Mammary carcinoma	0.9 ± 0.05	27 ± 5
6	Boxer	9	Mammary carcinoma	0.6 ± 0.03	21 ± 4
7	Welsh Springer Spaniel	9	Mammary carcinoma	1.6 ± 0.11	54 ± 8
8	English Springer Spaniel	10	Mammary adenoma	1.5 ± 0.14	35 ± 5
9	German Shepherd	7	Mammary carcinoma	0.7 ± 0.03	23 ± 3
10	Belgian Shepherd	9	UC	0.5 ± 0.01	17 ± 3
11	Riesenschnauzer	14	Mammary carcinoma	0.9 ± 0.02	30 ± 3
12	Boxer	11	Mammary adenoma	1.4 ± 0.1	27 ± 4
13	Boxer	7	Mammary carcinoma	1.2 ± 0.15	31 ± 5
14	Rottweiler	9	Mammary adenoma	0.5 ± 0.02	12 ± 3
15	German Shepherd	11	Mammary adenoma	0.8 ± 0.03	39 ± 6
16	Dachshund	9	Mammary carcinoma	0.9 ± 0.04	49 ± 5
17	Greyhound	12	UC	1.8 ± 0.18	28 ± 4
18	Rhodesian Ridgeback	11	Mammary adenoma	1.0 ± 0.06	17 ± 3
19	Rottweiler	10	Mammary adenoma	0.9 ± 0.05	28 ± 2
20	Boxer	8	UC	0.7 ± 0.04	9 ± 2
21	Rottweiler	10	Mammary carcinoma	0.9 ± 0.04	40 ± 8
22	Rottweiler	7	Mammary adenoma	0.7 ± 0.05	18 ± 5
23	German Shepherd	10	Mammary carcinoma	1.3 ± 0.08	31 ± 7
24	Golden Retriever	8	Mammary carcinoma	1.5 ± 0.12	43 ± 9
25	Flatcoated Retriever	6	Mammary adenoma	1.0 ± 0.09	28 ± 5
26	Mixed breed	6	Mammary carcinoma	1.1 ± 0.08	49 ± 10
27	Border Collie	14	Mammary carcinoma	1.6 ± 0.14	13 ± 4

^a^Mean values of three observations from a single measurement occasion. ^b^Mean values of two observations from two independent experiments. SD = standard deviation. Mammary tumors are classified as adenoma (benign), carcinoma (malignant), and UC = unclassified.

**Figure 3:**
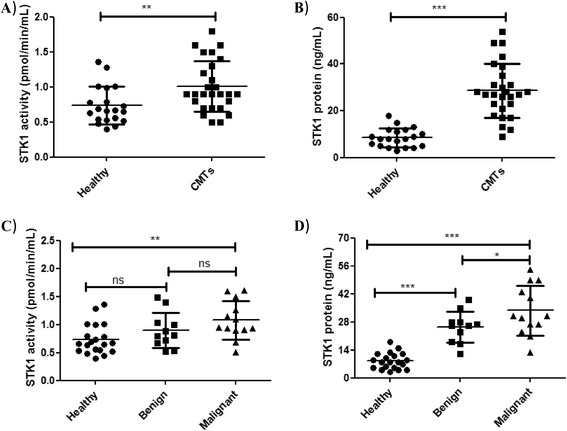
**Serum TK1 activity and TK1 protein concentrations in different clinical samples. (A)** Serum thymidine kinase 1 (sTK1) activity (pmol/min/mL) in sera from healthy dogs (•) and dogs with **mammary tumors (■).** The error bars represent means ± standard deviation (SD). **(B)** Serum TK1 protein levels in sera from healthy dogs (•) and dogs with canine mammary tumors (CMTs) (■). **(C)** Comparison of sTK1 activity in healthy dogs (•) and dogs with benign (■) and malignant (▲) CMTs. **(D)** Comparison of sTK1 protein levels in healthy dogs (•) and dogs with benign (■) and malignant (▲) mammary tumors. The error bars represent means ± standard deviation (SD).

**Figure 4:**
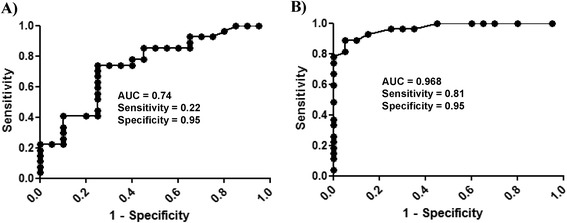
**Receiver operating characteristic (ROC) curve for the serum thymidine kinase 1 (sTK1) in healthy and canine mammary sera. (A)** sTK1 activity. **(B)** sTK1 protein assay results.

Significant differences were found in mean sTK1 activity (P = 0.007; Figure 3A) and sTK1 protein levels (P < 0.0001; Figure 3B) between healthy and CMT dogs. Significant correlations were also found between the sTK1 activity and sTK1 protein levels in healthy dogs (*r* = 0.52, P = 0.01) and CMTs (*r* = 0.41, P = 0.03). Canine mammary tumors were further classified as benign or malignant and the sTK1 protein and activity levels were determined in these subgroups and compared to those in healthy dogs. There was no significant difference in sTK1 activity in sera from healthy, benign, and malignant dogs (Figure 3C). However, a significant difference was found in sTK1 protein levels between these subgroups (Figure 3D). To evaluate the performance of the two different assays, ROC curve analysis for CMTs vs. healthy dogs was done. The results showed that TK1 activity assay had an area under the curve (AUC) of 0.74, P *=* 0.0048 (95% confidence interval (CI) 0.59–0.88), with a cutoff value of 1.32 pmol/min/mL, sensitivity of 0.22, and specificity of 0.95 (Figure 4A). The TK1 protein assay, however, had an AUC of 0.96, P < 0.0001 (95% CI 0.92–1.01). The sensitivity in this case was 0.81 and the specificity 0.95, using a cutoff value of 17.5 ng/mL (Figure 4B). The specific activity of sTK1 (nmol dTMP/min/mg of sTK of 26 kDa), based on the immunoaffinity assay and ^3^[H]-dThd activity measurements in healthy dogs, was 49 ± 21 nmol/min/mg, which is significantly higher (P < 0.0001) compared to sera from the CMT group (20 ± 11 nmol/min/mg). The higher sTK1 specific activity in healthy dogs (about 2.5-fold), compared to dogs with CMTs, means that there is a larger fraction of inactive TK1 polypeptide in case of CMTs.

### Molecular forms of thymidine kinase 1 in acute lymphocytic leukemia extract and sera

**Figure 5:**
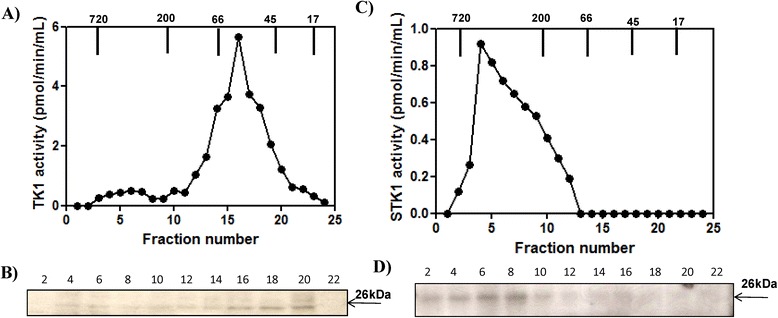
**Acute lymphocytic leukemia (ALL) cell extract and serum analyzed by Superose 12 column chromatography. (A)** The thymidine kinase 1 (TK1) activity in the fractions from the ALL cell extract (•). **(B)** Western blot analyses of the same fractions. **(C)** Serum TK1 (sTK1) activity in the fractions from the ALL dog (•). **(D)** Western blot analyses and sTK1 protein in the same fractions. Arrows indicate the elution position of the molecular weight (MW) markers.

A fresh blood sample collected from a dog with ALL and white blood cells were separated, as described in the Materials and Methods section. Cellular extract and sera from the same dog were applied to the Superose 12 column and the TK1 activity and protein levels in the fractions were determined. Native cellular TK1 activity eluted as major peak with molecular weights (MWs) of 40–66 kDa, but with a small peak and some enzyme activity in the fractions at the higher MW range (Figure 5A). Western blot analysis of cellular extract fractions showed a TK1 subunit (26 kDa) in the fractions eluted, corresponding to MWs of 40–66 kDa, with faint bands in the high MW region (Figure 5B). When analyzing sera from the same dog, about 90% of TK1 eluted as a peak in the high MW range corresponding to 200–720 kDa (Figure 5C). A TK1 polypeptide of 26 kDa was detected in fractions 1–12, but the band intensity did not correlate with the activity levels in these fractions (Figure 5D). These results indicate that active cellular TK1 isolated from ALL exists mainly as dimer, while active sTK1 from the same dog was found to occur as a high MW oligomer. A gel filtration experiment has been carried out with sera from another ALL dog and similar results were found (data not shown).

### Molecular forms of thymidine kinase 1 in mammary tumor extract and sera

**Figure 6:**
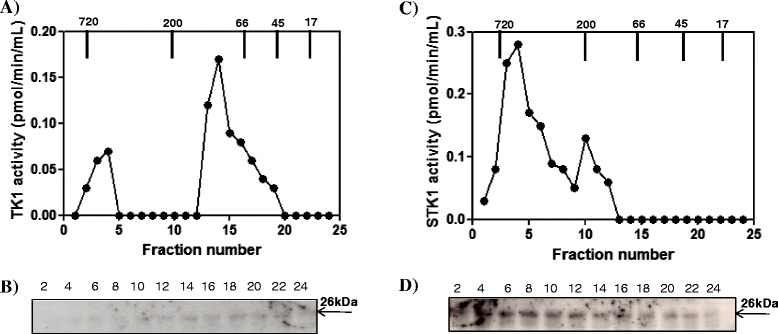
**Canine mammary tumor (CMT) (dog No. 23) tumor tissue extract and serum analyzed by Superose 12 column chromatography. (A)** Thymidine kinase 1 activity in the fraction from the CMT tissue extract (•). **(B)** Western blot analyses of the same fractions. **(C)** Thymidine kinase 1 activity in the fractions from the CMT serum (•). **(D)** Western blot analyses of the same fractions. Arrows indicate the elution position of the molecular weight (MW) markers. Numbers represent the fast protein liquid chromatography (FPLC) fractions.

Tissue extract prepared from a CMT patient (No. 23) was applied on the Superose 12 column and around 70% of total TK1 activity eluted as major peak with MWs of 40–100 kDa, but with a minor peak and some enzyme activity in the fractions corresponding to higher MW (Figure 6A). Western blot analysis of CMT extract showed a TK1 subunit (26 kDa) in the fractions corresponding to the peak and faint bands in the high MW region (Figure 6B). A serum sample from the same CMT dog was also analyzed and more than 95% of the total TK1 activity eluted in fractions 1–11, corresponding to the MW range 200–720 kDa (Figure 6C). Thymidine kinase 1 polypeptide of 26 kDa was detected in all fractions (Figure 6D) and there was no correlation with TK1 activity. These results indicate that sTK1 protein exists in multimeric forms in CMT (oligomers, dimers, and tetramers), many of them apparently inactive.

### Molecular forms of thymidine kinase 1 in healthy dog sera

**Figure 7:**
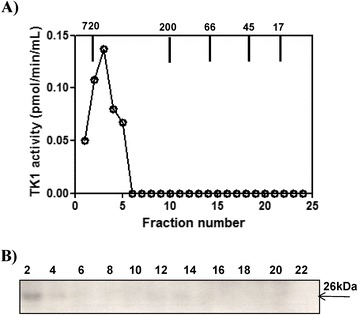
**Serum from a healthy dog (No. 5) analyzed by Superose 12 column chromatography. (A)** Serum thymidine kinase 1 (sTK1) activity in the fractions from the healthy dog (•). **(B)** Western blot analyses of sTK1 protein in the same fractions. Arrows indicate the elution position of the molecular weight (MW) markers. Numbers represent the fast protein liquid chromatography (FPLC) fractions.

**Figure 8:**
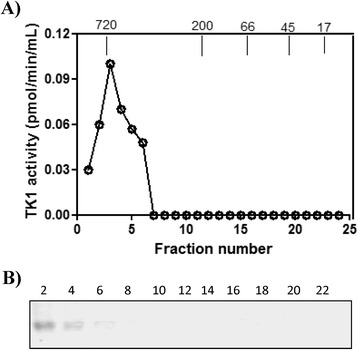
**STK1 profile in FPLC fractions of another healthy dog (sample no. 14, See Table** **1** **) (A)** **Serum thymidine kinase 1 (sTK1) activity in the fractions from the healthy dog (•).** **(B)** Western blot analyses of sTK1 protein in the same fractions. Arrows indicate the elution position of the molecular weight (MW) markers. Numbers represent the fast protein liquid chromatography (FPLC) fractions.

A serum sample from a healthy dog (No. 5) was subjected to size exclusion chromatography. Thymidine kinase 1 activity was recovered as a single peak in fractions 1–5, corresponding to a MW range of 500–720 kDa (Figure 7A). Western blot analysis showed a TK1 polypeptide of 26 kDa in the same fractions (Figure 7B). These results demonstrate that sTK1 exists as an active high MW oligomer in sera of healthy dogs. When we analyzed another healthy dog serum on superose 12 column, similar results were found (Figure 8).

## Discussion

Serum TK1 determinations have been used clinically for many years as they provide valuable information to guide anti-cancer therapy both in human and in veterinary medicine. Development of antibody-based assays has extended the use of TK1 in human breast cancer. Some studies in humans have shown that breast cancer sera contain high sTK1 protein levels, which can serve as prognostic marker for early stage detection [24,40,41]. A recent study in dogs with solid tumors has shown that TK1 protein assays are more sensitive compared to TK1 activity assays [37]. Mammary tumors occur more frequently in dogs than in other species and diagnosing patients during early stages of disease will be valuable clinically. In this study, we evaluated if sTK1 activity assay and TK1 protein assay can effectively differentiate CMT patients from healthy dogs.

The reason for comparing ALL and CMT was to show the difference in the molecular forms of TK1 in Hematological tumors (ALL) and solid tumors (CMT). In our last published study [38] we demonstrated the molecular forms of STK1 in an ALL patient and it was hence highly interesting to repeat these studies together with a characterization of the molecular forms of cellular (i.e. the tumor cells) TK1 from the same ALL patient. Furthermore, CMT are the most common tumors in older dogs and to develop an assay that can aid in the diagnosis of these types of tumors would be highly clinically valuable.

Even though sTK1 activity levels in CMTs significantly differ from levels in healthy dogs, there is a large overlap between levels in the two groups. The results presented here using sTK1 protein assays showed a larger difference and CMT patients had significantly higher sTK1 protein levels compared to healthy dogs. In this study, sTK1 protein assays were apparently able to differentiate benign from healthy samples as well as benign from the malignant forms of CMT, indicating that it may be possible to predict tumor progression by measuring sTK1 protein levels. Furthermore, the ROC curve analysis showed that the sTK1 protein assay had a sensitivity of 0.81, while the sTK1 activity assay showed a sensitivity of 0.22. Consequently, the sTK1 protein assay is most likely very valuable for establishing prognosis and early diagnosis in CMTs. Recently, expression analyses in CMTs have shown significant similarities to human breast cancer [42]. Consequently, it is suggested that these profiles can aid in prognosticating outcome [43,44]. However, these expression analyses are still complicated and most likely useful only as research tools. Moreover, they require specific, non-routine tissue preparation. A simple protein measurement in serum from a mammary tumor patient would be significantly easier to use and have a direct clinical implication. A natural next step in this research is to investigate if TK protein levels are correlated to, e.g., BRCA status in canine mammary tumors [9] and, if so, if TK protein level analysis is a suitable complement to more elaborate expression analyses of extirpated tumor tissue and is possible to perform before surgery. This could become a marker to gate patients into more, or less, aggressive surgical/medical interventions and could likely positively affect outcomes. It could also reduce surgical trauma in a fair portion of cases, which is ethically attractive. In the present study no correlation was tested between e.g. high/low grade in the CMT group because of low power. It is known that half of the malignant CMT (later) metastasize. Thus, it would be clinically highly beneficial if an early indication of the metastatic potential of the individual tumor could be made. The present study clearly indicates the need for prospective follow-up studies, where larger groups of CMTs are collected, with possibility to collect follow-up information on clinical outcome and compare histopathological grade and pre/post-op STK1 protein values to see if a decline in STK1 levels is seen post-op, further indicating the correlation between STK1 levels and CMT.

However, a lack of more detailed information about the molecular forms of sTK1 in dogs with tumor disease is a problem when it comes to using TK1 as a biomarker. In this study, we characterized cellular and serum forms of TK1. The cellular TK1 in ALL cells was found to exist mainly as active dimers, as reported previously [38,45], while sTK1 occurs as high MW oligomeric complexes. There was no direct correlation between sTK1 protein and activity levels, as shown previously [38], and this was apparently due to the different types of TK1 protein complexes, with varying specific activity. Active TK1 in CMT cell extracts was mainly found as tetramers, while sTK1 activity occurred predominantly as high MW oligomers. The 26 kDa TK1 protein levels were correlated to the activity in cell extract, but in case of sTK1, the 26 kDa polypeptide was detected in all fractions corresponding to a broad range of MWs. In sTK1 in healthy dogs, where the levels were low, both TK1 activity and protein were detected only in the high MW region. There is a correlation between sTK1 activity and protein levels in sera from healthy dogs (*r* = 0.52) and sera from dogs with CMTs (*r* = 0.41). This is most likely because activity assays measure the high MW complex of TK1, whereas the TK1 protein assays measure both active high MW and inactive lower MW forms. There was a difference in the mean specific activities of sTK1 from healthy dogs, dogs with CMT, and the dog with ALL, namely, 49 ± 21, 20 ± 10, and 760 ± 40 nmol/min/mg, respectively. The results obtained in this study are similar to those described earlier [37]. The specific activity of sTK1 was measured in a relatively large group of dogs with CMT and was found to be about half that of sTK1 in healthy dogs and about 2% of that observed in the ALL dog, which is very close to results observed with recombinant canine TK1 [38].

In this study, we have attempted to determine total tumor TK1 protein and activity levels as well as sTK1 levels in ALL and CMT dogs, using the same methods. There was a large difference in the ratio between the cellular and sTK1 total activity and 26 kDa TK1 protein levels in the ALL and CMT dogs (both about 50-fold). The reason for these large differences is most likely related to the differences in specific activities of the cellular and serum form of TK1 and this in turn is linked to the mechanisms involved in the accumulation and release of TK1 from hematologic and solid tumors. However, further studies are needed to clarify this question.

Normally, TK1 is present in relatively low concentrations in blood from healthy dogs. It is 5–20-fold in malignant diseases [37,38]. Therefore, sensitive laboratory methods are needed to obtain sufficient specificity and sensitivity. With the current available enzyme activity assays, this was achieved only for hematological tumors. These enzyme activity assays were not sufficient for detecting mammary tumors in dogs. However, sTK1 protein assays differentiate CMTs from healthy dogs more effectively and improve the clinical precision. The work described here will help to develop a reliable TK1 clinical test, which could be valuable in veterinary medicine. Furthermore, with the similarities to human mammary tumors, it will likely have comparative implications in breast cancer research.

## Conclusions

The results from this study show that TK1 protein assays provide more clinically relevant information on CMTs compared to TK1 activity assays. Size exclusion chromatography analysis demonstrates that sTK1 exists as a high MW complex form in dogs with ALL, healthy dogs, and dogs with CMT, with active enzyme. In addition, in sera from dogs with CMT, there is a large fraction of inactive TK1 in intermediate and low MW complexes. These results will help to develop better TK1 protein assays of value for human and veterinary medicine.

## Abbreviations

ALL: Acute lymphocytic leukemia
ANOVA: Analysis of variance
AUC: Area under the curve
BSA: Bovine serum albumin
CI: Confidence interval
CMT: Canine mammary tumor
CNBr: Cyanogen bromide
DNA: Deoxyribonucleic acid
dThd: Deoxythymidine
dTMP: Deoxythymidine monophosphate
DTT: Dithiothreitol
EDTA: Ethylenediaminetetraacetic acid
FCS: Fetal calf serum
FPLC: Fast protein liquid chromatography
HEPES: Hydroxyethyl-piperazineethane-sulfonic acid
MW: Molecular weight
PBS: Phosphate-buffered saline
ROC: Receiver operating characteristic
RPM: Revolution per minute
RPMI: Roswell Park Memorial Institute
SD: Standard deviation
SDS: Sodium dodecyl sulfate
SDS-PAGE: Sodium dodecyl sulfate polyacrylamide gel electrophoresis
sTK1: Serum thymidine kinase 1
TBS: Tris-buffered saline
TK: Thymidine kinase
TK1: Thymidine kinase 1
